# Hemodynamic correlates of transient cognitive impairment after transient ischemic attack and minor stroke: A transcranial Doppler study

**DOI:** 10.1177/1747493016661565

**Published:** 2016-07-26

**Authors:** Sara Mazzucco, Linxin Li, Maria A Tuna, Sarah T Pendlebury, Rose Wharton, Peter M Rothwell, on behalf of the Oxford Vascular Study

**Affiliations:** Nuffield Department of Clinical Neurosciences, University of Oxford, Oxford, UK

**Keywords:** Transcranial Doppler, blood flow velocity, cerebrovascular diseases, stroke, cognitive impairment, dementia

## Abstract

**Background and aims:**

Transient cognitive impairment (TCI) on the Mini Mental State Evaluation score is common after transient ischemic attack/minor stroke and might identify patients at increased risk of dementia. We aimed to replicate TCI using the Montreal Cognitive Assessment (MoCA), compare it with persistent Mild Cognitive Impairment (PMCI), and to determine whether global cerebral hemodynamic changes could explain transient impairment.

**Methods:**

Consecutive patients with transient ischemic attack/minor stroke (NIHSS ≤ 3) were assessed with the MoCA and transcranial Doppler ultrasound acutely and at 1 month. We compared patients with TCI (baseline MoCA < 26 with ≥ 2 points increase at 1 month), PMCI (MoCA < 26 with < 2 points increase), and no cognitive impairment (NCI; MoCA ≥ 26).

**Results:**

Of 326 patients, 46 (14.1%) had PMCI, 98 (30.1%) TCI, and 182 (55.8%) NCI. At baseline, TCI patients had higher systolic blood pressure (150.95 ± 21.52 vs 144.86 ± 22.44 mmHg, p = 0.02) and lower cerebral blood flow velocities, particularly end-diastolic velocity (30.16 ± 9.63 vs 35.02 ± 9.01 cm/s, p < 0.001) and mean flow velocity (48.95 ± 12.72 vs 54 ± 12.46 cm/s, p = 0.001) than those with NCI, but similar clinical and hemodynamic profiles to those with PMCI. Systolic BP fell between baseline and 1 month (mean reduction = 14.01 ± 21.26 mmHg) and end-diastolic velocity and mean flow velocity increased (mean increase = + 2.42 ± 6.41 and 1.89 ± 8.77 cm/s, respectively), but these changes did not differ between patients with TCI, PMCI, and NCI.

**Conclusions:**

TCI is detectable with the MoCA after transient ischemic attack and minor stroke and has similar clinical and hemodynamic profile to PMCI. However, TCI does not appear to be due to exaggerated acute reversible global hemodynamic changes.

## Introduction

Risk of dementia is increased on follow-up after transient ischemic attack (TIA) and stroke.^1^ Identifying early markers of increased risk of cognitive decline and dementia has the potential to inform the development of disease-modifying therapies and has clinical implications for counseling, long-term care planning, driving, and legal and financial issues.^2^ Mild cognitive impairment (MCI) precedes dementia in up to 80% of cases,^3^ and predictors of risk of conversion to dementia in MCI have been identified,^4^ including cerebrospinal fluid biomarkers,^5^ MRI hippocampal volumetry,^6^ and cerebral blood flow (CBF).^7^ However, more data are required on simple clinical predictors. For example, transient cognitive impairment (TCI) after TIA or minor stroke, first described using the Mini Mental State Evaluation (MMSE), might predict an increased risk of subsequent dementia on follow-up.^8^

TCI might therefore identify a subgroup of patients with exhausted cognitive reserve, but several issues remain to be resolved. First, TCI should be detectable using cognitive tests other than the MMSE. Compared with the MMSE, the Montreal Cognitive Assessment test (MoCA) has proved to be more sensitive and specific as a screening test for MCI,^9^ particularly after cerebrovascular events, and it incorporates executive and attentional tasks suitable for vascular cognitive impairment.^10^ We therefore aimed to determine the rate of TCI after TIA and stroke using the MoCA. Second, the cause of TCI is unknown, but could be related to transient global flow disturbances due to changes in blood pressure, endothelial function, or platelet activation in the acute phase after TIA or minor stroke. Cerebral hypoperfusion has been reported in the early stages of cognitive decline, using single-photon emission CT (SPECT)^11^ and carotid ultrasound,^12^ and transcranial Doppler (TCD) studies have documented reduced blood flow velocities (BFV) in the middle cerebral artery (MCA) in both dementia^13^ and MCI.^14^

We therefore hypothesized that TCI and MCI might have similar clinical and hemodynamic phenotypes and that TCI might be due to exaggerated but reversible global hemodynamic changes in the acute phase of TIAs and minor strokes.

## Methods

### Participants

This study was nested in the Oxford Vascular (OXVASC) Study, an ongoing population-based study of the incidence and outcome of all acute vascular events in a population of 92,728 individuals registered with 100 primary care physicians in nine practices in Oxfordshire, UK. Multiple methods of ascertainment are used for patients with TIA or stroke, as detailed elsewhere.^15^ These include a daily, rapid-access TIA/stroke clinic, to which participating physicians and the local emergency department refer all individuals with suspected TIA or minor stroke.^16^

Consecutive eligible patients attending the OXVASC rapid-access TIA/stroke clinic between November 2011 and April 2015 were enrolled in the present study. Patients with presumed TIA or minor stroke (NIHSS ≤ 3) were eligible if they were able to undergo cognitive assessment; had no pre-existing clinical diagnosis of dementia in the primary care records; had no disabling neurological deficit due to previous events; were willing and able to come back to clinic for a 1-month follow-up assessment; and had a temporal bone window suitable for ultrasound insonation.

### Procedures

Patients enrolled in the study were evaluated at two time-points, acutely, in the rapid-access TIA/stroke clinic, and at the 1-month follow-up visit. Patients were assessed by a neurologist or stroke physician and all presentations and investigations were reviewed by the senior study neurologist. During the acute clinical assessment, brain and vascular imaging were obtained, either 3T MRI with time-of-flight MRA of the intracranial vessels and a contrast enhanced MRA of the large neck arteries or brain CT with contrast enhanced CTA/Duplex ultrasound if MRI was contraindicated.

The MoCA was administered at initial assessment and was repeated at 1-month follow-up along with the MMSE. At both time points, TCD sonography (Doppler Box, Compumedics DWL, Singen, Germany) was performed by one of three experienced operators, who were unaware of the patient’s clinical presentation and results of cognitive assessment. MCA BFV was recorded with a handheld 2 MHz probe through temporal bone window at the depth that provided the best signal. Each session was stored in the hard disk of the TCD device for subsequent off-line analysis.

Secondary prevention treatment was started after the initial assessment and included aspirin (300 mg loading and then 75 mg daily), plus clopidogrel (300 mg loading dose and then 75 mg for 30 days) for high-risk patients; atorvastatin (40 mg daily); antihypertensive treatment (unless systolic blood pressure was below 130 mm Hg on repeated measurement), according to a standardized protocol: a combination of perindopril 5 mg and indapamide 1.25 mg followed by addition of amlodipine 5/10 mg, if necessary.

The OXVASC study and TCD assessment were approved by the local ethics committee and consent was obtained from all participants.

### Statistical analysis

To validate TCI using the MoCA, the mean change in MoCA score between baseline and follow-up was calculated on all recruited patients. MoCA scores were also divided into the 10 standard cognitive domains,^9^ which included visuoexecutive, naming, digit spam, attention, calculation, repetition, verbal fluency, abstract reasoning, recall, and orientation. Changes in each sub-score were calculated between baseline and follow-up.

For analyses of TCD parameters, patients with significant ICA or MCA stenosis (≥50%)^17^ on vascular imaging were excluded, as were patients who had recurrent stroke between baseline assessment and 1-month follow-up. However, to avoid any selection bias, patients recruited with what was considered initially to be a TIA, but in whom an alternative diagnosis was subsequently made on the basis of further investigation and follow-up were included in the main analysis, but sensitivity analyses excluding these cases were also done.

For further analysis of TCD data, cognitive patterns were classified as “No cognitive impairment” if MoCA score at baseline assessment was ≥ 26. Patients with MoCA score <26 were classified as either “Persistent mild cognitive impairment” (PMCI) if their MoCA score at 1 month did not increase by more than one point, or “TCI” if their score increased by ≥2 points. Demographic, clinical, cognitive, physiological characteristics, and risk factors of patients in these three groups were compared using χ^2^ test or ANOVA as appropriate. Degree of white matter changes (WMC) in the three groups was compared using the age-related WMC scale for both CT and MRI,^18^ rating five different regions in both hemispheres according to a 0–3 score. Total score was categorized as absent (0), mild (1–5), moderate (6–10), and severe (>10).

TCD measures of peak systolic velocity (PSV), end-diastolic velocity (EDV), mean flow velocity (MFV), and pulsatility index (PI) are given as a mean of the average of two measurements on each side; systolic blood pressure (SBP) and diastolic blood pressure (DBP) are given as mean of two measurements taken during the TCD procedure. Cerebrovascular resistance index (CVRi = mean blood pressure/MFV) was also calculated, consistently.

Analyses were repeated after stratification of the cohort in three age groups: <60, 60–70, and > 70 years of age. All analyses were done in SPSS version 22.

## Results

**Figure 1.**
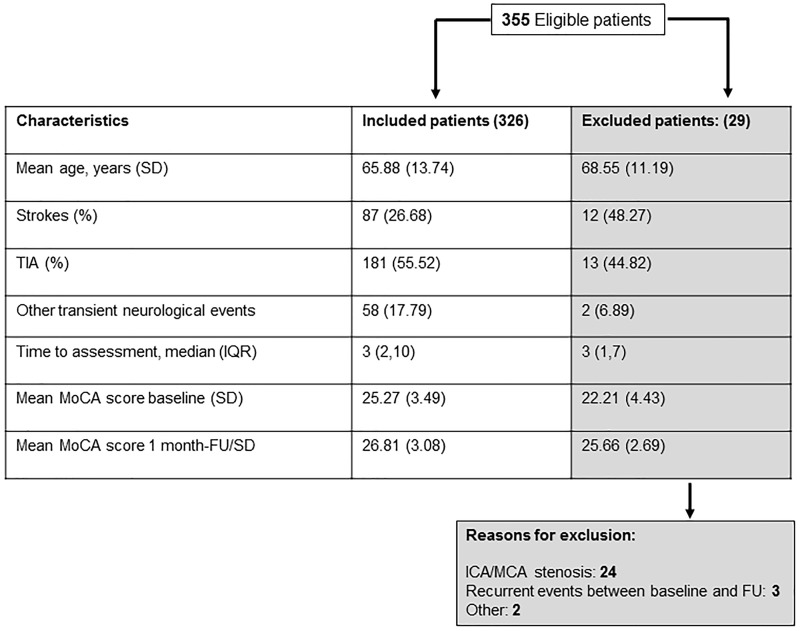
Characteristics of patients initially recruited who were included versus excluded from the primary analyses of physiological parameters. MoCA: Montreal Cognitive Assessment; ICA: internal carotid artery; MCA: middle cerebral artery; FU: follow-up.

Among all 355 eligible patients initially recruited (Figure 1), there was a significant overall increase in mean MoCA score between baseline and follow-up (mean change = 1.69, 95% CI = 1.43–1.95, p < 0.001). Mean/SD change was significant for the visuo-executive cognitive domain (+0.21/1, p < 0.001), naming (+0.05/0.34, p = 0.008), calculation (+0.09/0.65, p = 0.007), repetition (+0.11/0.72, p = 0.004), verbal fluency (+0.07/0.52, p = 0.008), abstract reasoning (0.17/0.60, p < 0.001), recall (+0.83/1.32, p < 0.001), and orientation (+0.13/0.63, p < 0.001) with non-significant increases in the digit span (+0.01/0.49) and attention (+0.02/0.31) domains.

Of this initially recruited group, 29 patients were excluded from the analyses of TCD parameters, mostly due either to identification of ≥ 50% ICA/MCA stenosis or a recurrent stroke prior to the 1-month follow-up (Figure 1). Of the remaining 326 patients, 268 (82.2%) had a clinically definite TIA or minor stroke and 58 (17.8%) had an eventual diagnosis of possible TIA or an alternative, most commonly migraine variant (N = 22). Results of sensitivity analyses of only patients with definite TIA/stroke are shown in Tables I and II of online supplement.

In the 326 eligible patients with repeat cognitive assessment and TCD at 1 month, mean SBP fell between baseline and 1-month (mean Δ SBP = −14.01, SD = 21.26 mmHg), whereas mean (SD) PSV, EDV, and MFV increased (ΔPSV = + 2.47(13.57) cm/s; ΔEDV = + 2.42 (6.41) cm/s; ΔMFV = + 1.89 (8.77) cm/s). Consistently, mean (SD) CVRi decreased by−0.29 (0.5).

Of these 326 patients, 46 (14.1%) had PMCI, 98 (30.1%) had TCI, and 182 (55.8%) had NCI (Table 1). Subtype of ischemic stroke for each group according to TOAST (Trial of ORG 10172 in Acute Stroke Treatment) are shown in Table IV of the supplement. Among patients who underwent MRI at baseline (269 out of 326, 82.5%), the frequency of DWI positive lesions did not differ between these groups (Table 1). Similarly, although time from the onset of the TIA or stroke to seeking medical attention varied (224 patients were seen within 7 days and 102 were seen after 7 days), cognitive group was unrelated to delay (Table 1). However, prevalence of TCI increased with age: 19% in the < 60 age group, 28% between 60 and 70, and 39% over 70 (p < 0.001).

**Table 1.** Clinical features of study patients stratified according to cognitive group

	PMCI (N = 46)	TCI (N = 98)	NCI (N = 182)	p
Age, mean (SD)	67.5 (15.23)	70.8 (13.16)	62.83 (12.85)	<0.001
*Presenting event*				
TIA	24 (52.2)	53 (54.1)	104 (57.1)	0.916
Stroke	14 (30.4)	29 (29.6)	44 (24.2)	
Other neurological events	8 (17.4)	16 (16.3)	34 (18.7)	
Time from event to assessment, median (IQR)	3 (1.75,9)	3.5 (2,11)	4 (2,10)	0.651
Time from assessment to follow-up, mean (SD)	38.7 (24)	37.7 (14.1)	34.5 (14.6)	0.13
Pre-morbid mRS 0–1	38 (84.4)	78 (84.8)	165 (93.8)	0.031
mRS at 1 month 0–1	34 (81)	63 (70)	128 (79)	0.20
BP lowering drugs started after baseline	25 (54.3)	57 (60)	100 (55.55)	0.733
History of hypertension	29 (64.44)	51 (53.76)	83 (45.85)	0.062
History of smoking	29 (67.44)	50 (53.76)	92 (51.68)	0.174
History of hyperlipidemia	16 (38.09)	35 (37.63)	54 (30.5)	0.403
History of diabetes mellitus	9 (20)	16 (16.84)	15 (8.28)	0.032
Low education	32 (69.56)	65 (66.32)	80 (43.95)	<0.001
DWI positive lesion on MR	6 (15.38)	18 (23.37)	30 (19.6)	0.583
Moderate to severe WMC	18 (38.7)	17 (17.6)	27 (15)	0.012
MoCA ≥ 26 at FU	10 (21.7)	62 (63.3)	170 (93.4)	<0.001

Data are number (%) unless otherwise stated.

PMCI: persistent mild cognitive impairment; TCI: transient cognitive impairment; NCI: no cognitive impairment; mRS: modified Rankin Scale; MoCA: Montreal Cognitive Assessment; BP: blood pressure; DWI: diffusion-weighted imaging; WMC: white matter changes using the age-related WMC scale for both CT and MRI.

When compared with NCI, TCI patients were older (p < 0.001), had higher pre-morbid mRS (p = 0.035), were more likely to be diabetic (p = 0.028), and to have a low education (p < 0.001) (Table 1). The TCI group showed higher mean SBP and lower MCA BFVs (Table 2) at baseline assessment, mainly with lower MFV, EDV, and higher PI and CVRi. These differences were also present at 1-month follow-up (Table 2) and were most marked in patients aged ≥ 60 years (Table III of online supplement). However, the changes in SBP, DBP, EDV, MFV, and CVRi between baseline and FU were similar in the two groups (Table 2).

**Table 2.** Comparison between physiological, hemodynamic, and cognitive variables in patients with TCI vs NCI at baseline and 1-month follow-up.

Mean (SD)	TCI (N = 98)	NCI (N = 182)	p
Baseline			
SBP baseline	150.95 (21.52)	144.86 (22.44)	0.02
DBP baseline	80.58 (10.14)	81.85 (11.35)	0.35
PSV baseline	78.75 (18.07)	84.70 (18.46)	0.01
EDV baseline	30.16 (9.63)	35.02 (9.01)	<0.001
MFV baseline	48.95 (12.72)	54 (12.46)	0.001
PI baseline	1.01 (0.22)	0.93 (0.2)	0.001
CVRi baseline	2.29 (0.74)	2.03 (0.62)	0.002
MoCA baseline	21.87 (3.17)	27.65 (1.21)	<0.001
1-month follow-up			
SBP FU	136.82 (18.22)	131.26 (18.13)	0.01
DBP FU	73.56 (9.11)	73.96 (9.44)	0.73
% SBP Diff	−8.28 (13.71)	−8.2 (13.23)	0.96
% DBP Diff	−7.69 (13.49)	−8.78 (11.44)	0.47
PSV FU	81.86 (16.32)	86.39 (19.47)	0.05
EDV FU	32.58 (9.68)	37.17 (9.48)	<0.001
MFV FU	51.19 (12.37)	55.7 (13.07)	0.005
PI FU	0.98 (0.22)	0.89 (0.2)	<0.001
CVRi FU	1.94 (0.52)	1.77 (0.49)	0.008
%PSV Diff	7.6 (20.36)	3.65 (17.14)	0.09
%EDV Diff	12.21 (28.84)	8.53 (22.10)	0.23
%MFV Diff	6.66 (19.24)	4.57 (18.49)	0.37
%PI Diff	−2.18 (12.92)	−3.16 (14.85)	0.58
%CVRi Diff	−12.23 (18.36)	−10.19 (18.78)	0.38
MoCA FU	25.8 (2.9)	28.34 (1.74)	<0.001
MoCA Diff	3.93 (1.77)	0.68 (1.73)	<0.001
MMSE FU	26.49 (2.62)	28.51 (1.92)	<0.001

TCI: transient cognitive impairment; NCI: no cognitive impairment; FU: follow-up; SBP: systolic blood pressure (mmHg); DBP: diastolic blood pressure (mmHg); PSV: peak systolic velocity (cm/s); EDV: end-diastolic velocity (cm/s); MFV: mean flow velocity (cm/s); PI: pulsatility index; CVRi: cerebrovascular resistance index; MoCA: Montreal Cognitive Assessment; % Diff: percent change between baseline and follow-up; MoCA Diff: MoCA score change between baseline and follow-up; MMSE: Mini Mental State Evaluation.

In contrast, patients with TCI did not differ from patients with PMCI in age (p = 0.19), pre-morbid disability (p = 0.61), low education (p = 0.43), or other risk factors (Table 1). Patients with TCI and PMCI also had similar physiological profiles in terms of BP and TCD parameters at baseline (Table 3). By definition, cognitive function was improved at 1 month in the TCI group (mean/SD ΔMoCA = 3.93/1.78), particularly in recall (Δ = 1.50/1.35), visuospatial domain (Δ = 0.70/1.16) and abstraction (Δ = 0.42/0.69), but changes in BP and TCD parameters did not differ from those in patients with PMCI and NCI (Tables 2 and 3). These findings did not change after excluding 36 (37%) patients with TCI in whom the MoCA at 1-month was improved but nevertheless remained < 26 (data not shown).

**Table 3.** Comparison between physiological, hemodynamic, and cognitive variables in patients with PMCI vs TCI at baseline and 1-month follow-up

Mean (SD)	PMCI (N = 46)	TCI (N = 98)	p
Baseline			
SBP baseline	149.56 (22.56)	150.94 (21.52)	0.724
DBP baseline	80.4 (10.25)	80.58 (10.14)	0.919
PSV baseline	79.88 (18.86)	78.75 (18.07)	0.73
EDV baseline	30.82 (10.04)	30.16 (9.63)	0.705
MFV baseline	49.14 (12.57)	48.95 (12.72)	0.933
PI baseline	1.01 (0.23)	1.01 (0.22)	0.959
CVRi baseline	2.26 (0.72)	2.29 (0.74)	0.822
MoCA baseline	23.11 (2.77)	21.87 (3.17)	0.025
1-month follow-up			
SBP FU	134.14 (16.82)	136.82 (18.22)	0.401
DBP FU	72.9 (8.38)	73.56 (9.11)	0.677
% SBP Diff	−9.05 (12.98)	−8.28 (13.71)	0.749
% DBP Diff	−8.46 (11.44)	−7.69 (13.49)	0.737
PSV FU	81.80 (18.36)	81.86 (16.32)	0.98
EDV FU	33.73 (12.07)	32.58 (9.68)	0.547
MFV FU	51.01 (13.99)	51.19 (12.37)	0.939
PI FU	0.99 (0.24)	0.98 (0.22)	0.951
CVRi FU	1.97 (0.59)	1.94 (0.52)	0.73
% PSV Diff	6.41 (22.23)	7.60 (20.36)	0.756
%EDV Diff	11.79 (26.6)	12.21 (28.84)	0.934
%MFV Diff	6.67 (19.24)	5.65 (22.63)	0.78
% PI Diff	−1.66 (16.50)	−2.18 (12.92)	0.839
% CVRi Diff	−10.25 (20.86)	−12.23 (18.36)	0.567
MoCA FU	22.93 (3.36)	25.8 (2.9)	<0.001
MoCA Diff	−0.17 (1.61)	3.93 (1.77)	<0.001
MMSE FU	25.24 (2.8)	26.49 (2.62)	0.012

PMCI: persistent mild cognitive impairment; TCI: transient cognitive impairment; FU: follow-up; SBP: systolic blood pressure (mmHg); DBP: diastolic blood pressure (mmHg); PSV: peak systolic velocity (cm/s); EDV: end-diastolic velocity (cm/s); MFV: mean flow velocity (cm/s); PI: pulsatility index; CVRi: cerebrovascular resistance index; MoCA: Montreal Cognitive Assessment; % Diff: percent change between baseline and follow-up; MoCA Diff: MoCA score change between baseline and follow-up; MMSE: Mini Mental State Evaluation.

## Discussion

This is the largest study, to our knowledge, of longitudinal changes of cognitive performance soon after a TIA/minor stroke. We showed an overall improvement in cognition between baseline assessment and 1-month follow-up, confirming that reversible cognitive impairment can be demonstrated after minor cerebral ischemic events using the MoCA, which is more sensitive and specific than MMSE as a screening test for MCI^9^ and for vascular cognitive impairment.^10^ We have also shown that patients with TCI have similar clinical and global hemodynamic characteristics to those with persisting MCI, but we were not able to explain resolution of TCI on the basis of improved MCA BFVs at 1-month follow-up.

Patients with TCI would, by definition, have been classified as cognitively impaired when first seen in the emergency clinic, scoring less than 26 on the MoCA. However, 63% of such patients scored ≥ 26 at the 1-month follow-up visit. Improvement was seen mainly in recall, as previously demonstrated with the MMSE,^8^ but also in the visuospatial and abstraction domain, confirming previous results with the MoCA,^19^ which is more sensitive to visuo-executive and attentional domains.^10^ These findings support the advice given in some countries that temporary abstaining from driving, and perhaps from other cognitively complex tasks, is advisable after a TIA/minor stroke.

Notwithstanding the improvement in the cognitive performance, TCI patients displayed a functional profile, risk factors, and MCA hemodynamic indices similar to PMCI patients, and this applied also when restricting the analysis only to the 63% of TCI patients with ≥ 26 MoCA score at 1 month. That is, patients with TCI who would have been classified as cognitively unimpaired at the 1-month follow-up visit had MCA perfusion indices similar to patients with persistent cognitive impairment. Previous data from the OXVASC study showed that TCI patients were more prone to develop cognitive decline and overt dementia on long-term follow-up,^8^ and further follow-up of the current cohort will confirm whether lower MCA BFVs predict cognitive decline.^14^

TCI could be an early manifestation of MCI in patients who are still usually performing within a normal cognitive range, but who have very limited cognitive reserve. Preclinical stages of dementia are associated to lower MCA MFVs,^13,14^ and higher PI could predict progression from MCI to overt dementia.^20^ However, we could not find any relation between the transient cognitive deterioration and changes in global cerebral hemodynamic variables. SBP and DBP decreased equally across our three cognitive groups between baseline and follow-up, probably as a result of antihypertensive treatment (Tables 2 and 3), with similar increases in cerebral BFVs.^21^ Moreover, as the frequency of TCI did not decrease with time since TIA or stroke, it is unlikely that an acute decrease in cerebral perfusion secondary to the cerebral vascular event could be the cause of transient of cognitive perturbation.

Consistent with previous reports,^8,18^ TCI patients were older than patients with no cognitive impairment. However, TCI patients were in the same age range as patients with PMCI. Cognition, BP, and cerebral flow velocities are all strongly related to age.^22^ When our patients were stratified by age, the proportion with TCI increased with age, and the differences in global hemodynamic features between patients with TCI and NCI became more evident (Table III of online supplement). Moreover, the differences were most evident in MCA EDV and MFV, which best reflect vascular resistance in cerebral circulation.^23^

Our study has some limitations. First, the definition of TCI as a two or more point improvement in MoCA score has not yet been widely standardized. However, it was based on previous evidence that changes in MoCA scores between evaluations 1 month apart averaged 0.9 points,^9^ and is also consistent with the only previous study on MoCA changes soon after TIA/minor stroke.^18^ Second, we do not yet have long-term follow-up to confirm our previous observation that TCI patients are more prone to develop permanent cognitive impairment.^8^ Third, we did not do any test of anxiety or depression at baseline or 1-month follow-up. TCI could, at least partly, be a non-specific transient perturbation in cognition in patients with exhausted cognitive reserve related to anxiety rather than being specifically caused by the cerebrovascular event, particularly in the acute assessment in a hospital setting with multiple unfamiliar investigations. A role for anxiety might also explain why the frequency of TCI was independent of the time interval between symptom-onset and baseline assessment. Fourth, TCD, measuring BFV, does not provide absolute quantification of CBF. However, MCA BFV indices are strongly related to cerebral peripheral resistance, in particular EDV, which reliably reflects changes in perfusion,^23^ PI, which is also a marker of small vessel disease,^24^ and CVRi, which reflects the relationship between blood pressure and CBF.^25^

Fifth, our hypothesis was that TCI could be caused by transient global flow disturbances in the acute phase of TIAs and minor strokes, but we couldn’t find any global hemodynamic change specifically associated to TCI. However, our TCD study cannot exclude focal hemodynamic abnormalities. Further studies with high sensitivity MRI could address this issue looking at focal perfusion abnormalities. Last, we have not investigated vascular territories other than the MCA like posterior circulation. Therefore, we cannot exclude that hemodynamic changes in other territories are related to cognitive changes in the TCI group.

## Conclusions

In conclusion, TCI is detectable with the MoCA after TIA and minor stroke and affected patients have similar clinical and hemodynamic profiles to those with PMCI. However, we were not able to demonstrate any exaggerated acute reversible changes in global hemodynamic indices in the TCI group that might have explained the transient cognitive dysfunction. Nevertheless, it is important that clinicians are aware of the possibility of TCI in patients with TIA and minor stroke, particularly in relation to the recall of advice given in the acute phase and the capacity to perform complex tasks.

## Supplementary Material

Supplementary material
