# Supplementary material for: Hemodynamic correlates of transient cognitive impairment after transient ischemic attack and minor stroke: A transcranial Doppler study
Source: Int J Stroke. 2016 Jul 26;11(9):978–86. doi: 10.1177/1747493016661565 (PMC5490782; doi:10.1177/1747493016661565)
Supplement: Supplementary material [file WSO661565.pdf]

Haemodynamic correlates of transient cognitive impairment after TIA and minor stroke: a transcranial Doppler study.

ONLINE SUPPLEMENT

**Baseline**			
**Mean (SD)**	**TCI (N=82)**	**NCI (N=148)**	**p**
**SBP baseline**	150.74 (22.13)	146.3 (22.03)	0.15
**DBP baseline**	80.37 (10.25)	82.43 (11.44)	0.18
**EDV baseline**	29.21 (8.76)	34.4 (9.32)	**<0.001**
**MFV baseline**	47.65 (11.61)	53.28 (12.83)	**0.001**
**PI baseline**	1.03 (0.22)	0.95 (0.21)	**0.007**
**MoCA baseline**	21.89 (3.25)	27.6 (1.17)	**<0.001**
**1 month Follow-up**			
**SBP FU**	135.95 (18.06)	130.31 (17)	**0.02**
**DBP FU**	72.73 (9.04)	73.35 (9.07)	0.62
**% SBP Diff**	-8.56 (14.5)	-9.77 (12.72)	0.51
**% DBP Diff**	-8.36 (14.31)	-10.21 (10.68)	0.27
**EDV FU**	31.38 (8.57)	36.74 (9.74)	**<0.001**
**MFV FU**	49.54 (11.27)	54.89 (13.39)	**0.002**
**PI FU**	0.99 (0.22)	0.9 (0.21)	**0.001**
**%EDV Diff**	12.1 (30.22)	9.64 (23.7)	0.5
**%MFV Diff**	6.07 (19.58)	4.61 (19.36)	0.59
**MoCA FU**	25.79 (2.96)	28.37 (1.61)	**<0.001**
**MoCA Diff**	3.9 (1.81)	0.77 (1.64)	**<0.001**
**MMSE FU**	26.51 (2.42)	28.59 (1.98)	**<0.001**

Table I: Sensitivity analysis; comparison between physiological, hemodynamic, and cognitive variables in transient cognitive impairment (TCI) vs no cognitive impairment (NCI) in TIAs and strokes only. Abbreviations: TCI = transient cognitive impairment; NCI= no cognitive impairment; FU= Follow-up; SBP= Systolic Blood Pressure (mmHg); DBP= Diastolic Blood Pressure (mmHg); EDV: End-Diastolic Velocity (cm/sec); MFV= Mean Flow Velocity (cm/sec); PI= Pulsatility Index; MoCA= Montreal Cognitive Assessment; % Diff= percent change between baseline and follow-up; MoCA Diff= MoCA score change between baseline and follow-up; MMSE= Mini Mental State Evaluation.

Baseline			
**Mean (SD)**	**TCI (N=82)**	**PMCI (N=38)**	**p**
**SBP baseline**	150.74 (22.13)	149.24 (23.71)	0.74
**DBP baseline**	80.37 (10.25)	80.71 (10.63)	0.87
**EDV baseline**	29.21 (8.76)	31.19 (10.68)	0.29
**MFV baseline**	47.65 (11.61)	49.38 (13.35)	0.47
**PI baseline**	1.03 (0.22)	1.00 (0.25)	0.61
**MoCA baseline**	21.89 (3.25)	23.05 (2.96)	0.06
**1 month Follow-up**			
**SBP FU**	135.95 (18.06)	132.93 (16.74)	0.39
**DBP FU**	72.73 (9.04)	72.96 (8.59)	0.89
**% SBP Diff**	-8.56 (14.5)	-9.43 (13.94)	0.76
**% DBP Diff**	-8.36 (14.31)	-8.61 (12.29)	0.93
**EDV FU**	31.38 (8.57)	35.04 (12.42)	0.07
**MFV FU**	49.54 (11.27)	52.26 (14.47)	0.26
**PI FU**	0.99 (0.22)	0.98 (0.25)	0.62
**%EDV Diff**	12.1 (30.22)	15.48 (27.32)	0.57
**%MFV Diff**	6.07 (19.58)	8.07 (23.49)	0.63
**MoCA FU**	25.79 (2.96)	22.87 (3.5)	**<0.001**
**MoCA Diff**	3.9 (1.81)	-0.18 (1.66)	**<0.001**
**MMSE FU**	26.51 (2.42)	25.08 (3)	**0.007**

Table II: sensitivity analysis; comparison between physiological, hemodynamic, and cognitive variables in transient cognitive impairment (TCI) vs persistent mild cognitive impairment (PMCI), including TIAs and strokes only. Abbreviations: PMCI= persistent mild cognitive impairment; TCI= transient cognitive impairment; FU= Follow-up; SBP= Systolic Blood Pressure (mmHg); DBP= Diastolic Blood Pressure (mmHg); EDV: End-Diastolic Velocity (cm/sec); MFV= Mean Flow Velocity (cm/sec); PI= Pulsatility Index; MoCA= Montreal Cognitive Assessment; % Diff= percent change between baseline and follow-up; MoCA Diff= MoCA score change between baseline and follow-up; MMSE= Mini Mental State Evaluation.

Age <60			
**Mean /SD**	**TCI (N=19)**	**NCI (N=71)**	**p**
**SBP baseline**	138.13 (12.93)	136.64 (21.11)	0.77
**EDV baseline**	42.61 (8.56)	40.01 (7.69)	0.2
**MFV baseline**	63.22 (12.6)	58.47 (11.11)	0.11
**PI baseline**	0.8/0.08)	0.82/0.11	0.49
**%EDV Diff**	2.66 (16.15)	3.96 (18.49)	0.78
**MoCA Diff**	3.79 (1.31)	0.89 (1.6)	**<0.001**

60≤Age ≤70			
**Mean /SD**	**TCI (N=23)**	**NCI (N=57)**	**p**
**SBP baseline**	147.84 (18.5)	145.39 (20.54)	0.62
**EDV baseline**	30.1 (6.65)	34.81 (8.59)	**0.02**
**MFV baseline**	49.09(11.03	53.76 (13.53)	0.146
**PI baseline**	0.97 (0.16)	0.92 (0.16)	0.29
**%EDV Diff**	10.01 (20.57)	10.83 (22.07)	0.87
**MoCA Diff**	3.49 (1.41)	0.51 (1.59)	**<0.001**

Age >70			
**Mean /SD**	**TCI (N=56)**	**NCI (N=54)**	**p**
**SBP baseline**	156.57 (23.07)	155.1 (22.09)	0.73
**EDV baseline**	25.95 (7.11)	28.69 (6.86)	**0.04**
**MFV baseline**	44.05 (9.48)	48.37 (10.7)	**0.02**
**PI baseline**	1.11 (0.21)	1.07 (0.24)	0.46
**%EDV Diff**	16.44 (34.17)	12.07 (25.56)	0.45
**MoCA Diff**	4.16 (2.01)	0.59 (2.01)	**<0.001**

Table III: age-stratified comparison between physiological, hemodynamic, and cognitive variables in transient cognitive impairment (TCI) and no cognitive impairment (NCI). Abbreviations: TCI= transient cognitive impairment; NCI = no cognitive impairment; SBP= Systolic Blood Pressure (mmHg); EDV: End-Diastolic Velocity (cm/sec); MFV= Mean Flow Velocity (cm/sec); PI= Pulsatility Index; % Diff= percent change between baseline and follow-up; MoCA Diff= MoCA score change between baseline and follow-up.

TOAST classification N (%)	PMCI (N=38)	TCI (N=81)	NCI (N=148)	P
CE	5 (12.9)	15 (17.9)	32 (21.5)	0.89
LAD	2 (6.5)	6 (7.5)	11 (7.5)	
SVD	6 (16.1)	12 (14.9)	15 (10.3)	
Other/Ukn/Mult/Und	25 (64.5)	49 (59.7)	90 (60.8)	

Table IV: TOAST classification for Strokes and TIAs included in the analysis (Other neurological events not included in etiological classification). Abbreviations: PMCI = persistent mild cognitive impairment; TCI = transient cognitive impairment; and NCI = no cognitive impairment; TOAST= Trial of ORG 10172 in Acute Stroke Treatment; CE= cardio-embolic; LAD= Large Artery Disease; SVD= Small Vessel Disease; Ukn= Unknown etiology; Mult= Multiple etiology; Und= Undetermined etiology. Numbers for each group are for ischemic strokes and TIAs only.

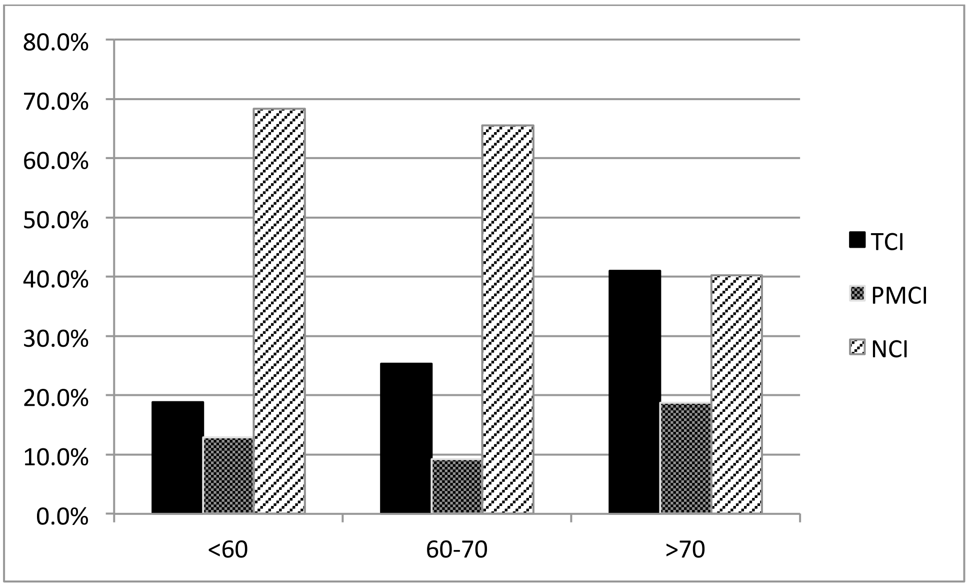

Figure A: Age-stratified proportion of patients with transient cognitive impairment (TCI), persistent mild cognitive impairment (PMCI), and no cognitive impairment (NCI).

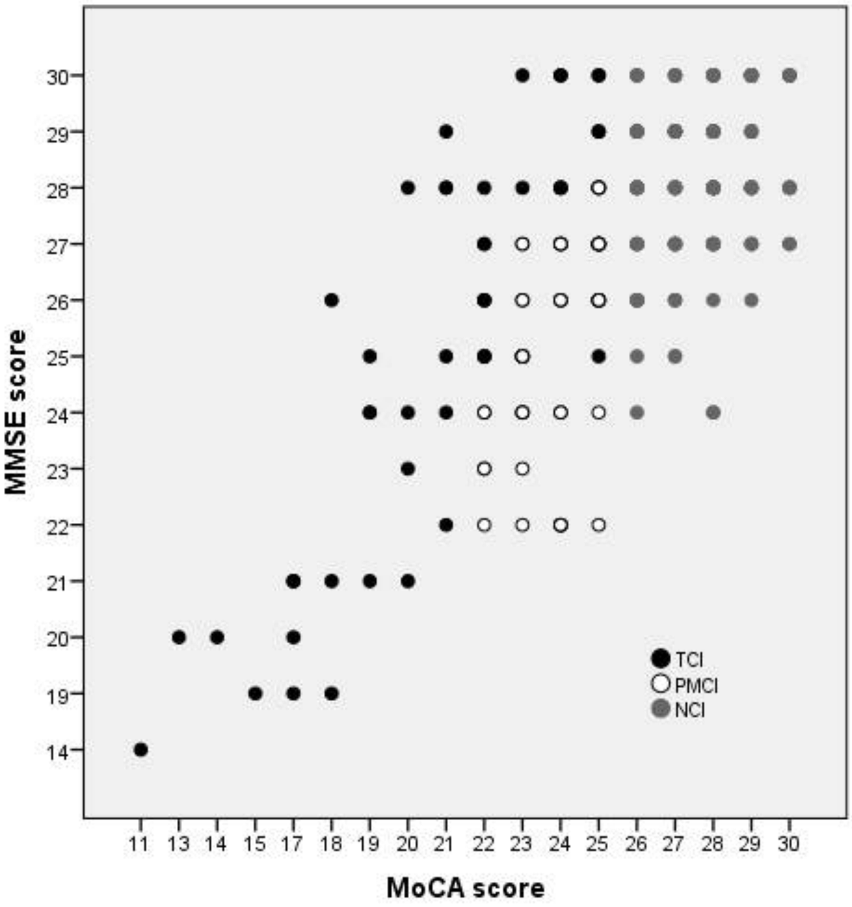

Figure B1: Correlation between MoCA and MMSE score in patients with transient cognitive impairment (TCI) according to the MoCA definition (black dots), persistent mild cognitive impairment (PMCI) (white dots), and no cognitive impairment (NCI) (grey dots) at baseline assessment. The R coefficient is 0.744, p<0.001.

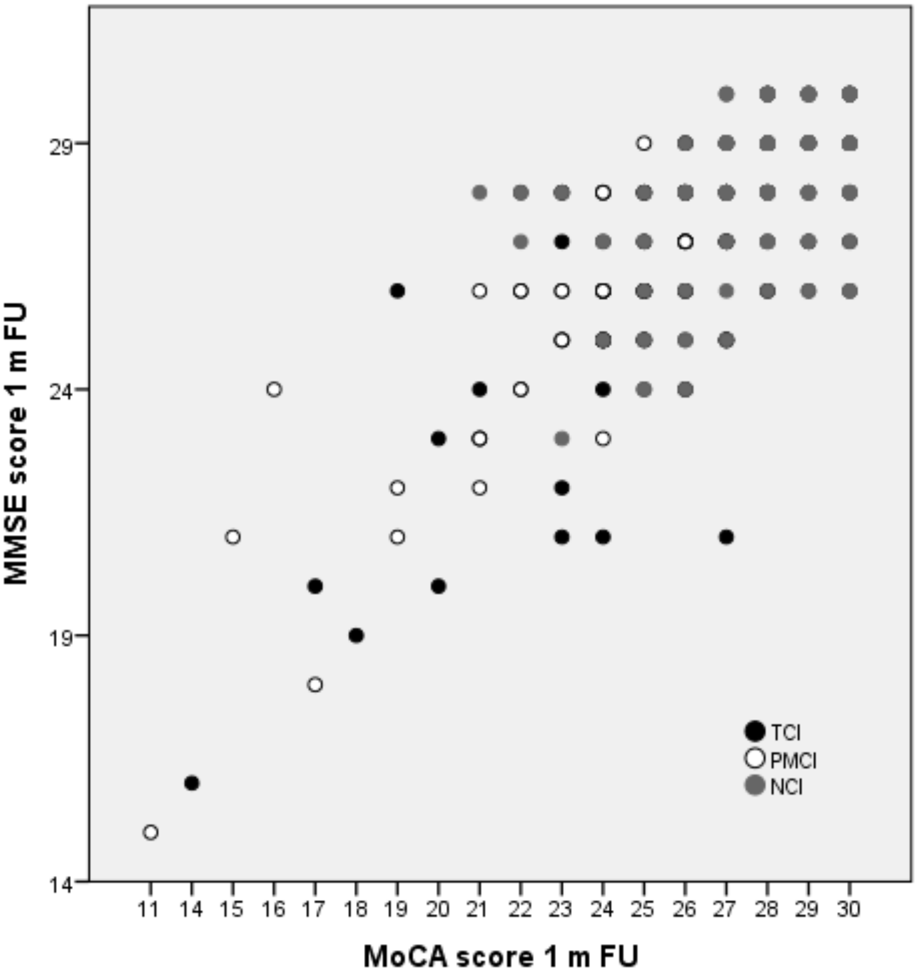

Figure B2: Correlation between MoCA and MMSE score in patients with transient cognitive impairment (TCI) according to the MoCA definition (black dots), persistent mild cognitive impairment (PMCI) (white dots), and no cognitive impairment (NCI) (grey dots) at one month follow-up visit. The R coefficient is 0.795, p<0.001.
